# A Systematic Review and Meta-Analysis on Aerobic Fitness Dynamics in Post-COVID-19 Athletes: Implications in the Return-to-Play Performance

**DOI:** 10.3390/sports13020040

**Published:** 2025-02-05

**Authors:** Lucas Rafael Lopes, Rui Medeiros, Valéria Tavares, Francisca Dias, Marcus Vinícius Galvão Amaral, Rodrigo Araújo Goes, João Antonio Matheus Guimarães, Jamila Alessandra Perini

**Affiliations:** 1 Research Laboratory of Pharmaceutical Sciences (LAPESF), Pharmacy Department, Rio de Janeiro State University (UERJ), Rio de Janeiro 23070-200, Brazil; lopes.rlucas02@gmail.com; 2 Program of Post-Graduation in Public Health and Environment, National School of Public Health, Oswaldo Cruz Foundation (Fiocruz), Rio de Janeiro 21041-210, Brazil; 3 Molecular Oncology and Viral Pathology Group, Research Center of IPO Porto (CI-IPOP), Pathology and Laboratory Medicine Department, Clinical Pathology SV,RISE@CI-IPOP (Health Research Network), Portuguese Oncology Institute of Porto (IPO Porto), Porto Comprehensive Cancer Centre (Porto.CCC), 4200-072 Porto, Portugal; ruimedei@ipoporto.min-saude.pt (R.M.); valeria.tavares@ipoporto.min-saude.pt (V.T.); francisca.carvalho.dias@ipoporto.min-saude.pt (F.D.); 4 Faculty of Medicine, University of Porto (FMUP), 4200-072 Porto, Portugal; 5 ICBAS—Instituto de Ciências Biomédicas Abel Salazar, Universidade do Porto, 4050-313 Porto, Portugal; 6 Research Department, Portuguese League Against Cancer (NRNorte), 4200-172 Porto, Portugal; 7 Faculty of Health Sciences, Fernando Pessoa University, 4200-150 Porto, Portugal; 8 Research Division, National Institute of Traumatology and Orthopaedics (INTO), Rio de Janeiro 20940-070, Braziljmatheusguimaraes@gmail.com (J.A.M.G.)

**Keywords:** COVID-19, athletes, performance, maximal oxygen uptake, aerobic fitness

## Abstract

Maximal oxygen uptake ($\dot{V}$O_2_max) assesses athletic performance; however, its values are inconsistent in post-COVID-19 athletes. This study aimed to analyze the dynamics of $\dot{V}$O_2_max in post-COVID-19 athletes. Observational studies were identified by screening the PubMed database published up to 17 July 2023. The initial electronic search found 320 studies. Of these, 26 employing the cardiopulmonary exercise test (CPET) to assess aerobic fitness were selected. Of the 2625 pooled athletes, 1464 were infected and considered as the post-COVID-19 group, either asymptomatic or symptomatic, while the remaining 1161, who were uninfected or had $\dot{V}$O_2_max results prior to infection, were defined as the infection-free group. Age and $\dot{V}$O_2_max were differently distributed between post-COVID-19 athletes and those without infection (*p* = 0.03 in both). Persistent symptoms athletes had 8 mL/Kg/min lower $\dot{V}$O_2_max than those without infection (*p* = 0.04). In addition, post-infected athletes who underwent CPET after 12 weeks showed a significant reduction of 2.9 mL/Kg/min in $\dot{V}$O_2_max according to the increase in body mass index (BMI). The pooled analysis showed that aerobic fitness was reduced in athletes post-COVID-19. $\dot{V}$O_2_max was negatively correlated with BMI in those who underwent CPET at 12 weeks, suggesting that symptoms persist beyond 12 weeks, affecting return-to-play.

## 1. Introduction

Coronavirus disease 2019 (COVID-19) has affected approximately 775.2 million people worldwide over three years (updated 24 March 2024). Even with 67% of the total global population vaccinated with a complete primary series and 32% with at least one booster dose of a COVID-19 vaccine, 42,388 individuals were infected from January to March 2024 [1]. This becomes a concern with the occurrence of major events, such as the Paris 2024 Olympic Games, where interaction between individuals from different countries may increase the number of cases and generate negative public health consequences, such as sequelae of infection [2]. Post-COVID-19 syndrome consists of persistent symptoms, including but not limited to breathlessness, increased oxygen requirements, post-viral cough, and cardiovascular muscular change, which can manifest for more than 12 weeks after the infection period [3,4]. Recent studies have shown that even asymptomatic and those with mild or moderate symptoms during acute infection may suffer post-COVID sequelae when returning to their training [5,6,7,8,9,10,11,12,13]. Indeed, the prevalence of persistent symptoms in the general population ranges from 8 to 45% [14], whereas in competitive athletes, it is around 1.2–8.3% [15]. However, small reductions in functional exercise capacity or cognitive achievement may contribute to their reduced athletic performance [16]. Current recommendations for return-to-play after the SARS-CoV-2 infection course are based on the presence of symptoms, cardiac and respiratory examinations, and exercise tolerance testing [17].

Cardiopulmonary exercise testing (CPET) is a useful tool for assessing cardiorespiratory fitness and exercise capacity in post-COVID athletes. It evaluates aerobic and anaerobic delivery limits for adenosine triphosphate (ATP) resynthesis, involving the cardiovascular, musculoskeletal, and pulmonary systems [18,19]. The main parameter measured from CPET is maximal oxygen uptake ($\dot{V}$O_2_max), which reflects aerobic fitness during exercise-induced stress [19]. Although studies have shown that COVID-19 often presents asymptomatically or with mild symptoms in elite athletes [5,6,7,9,11,12,20,21,22,23], others have shown that post-COVID athletes have lower performance levels with reduced $\dot{V}$O_2_max values compared to their counterparts, despite having recovered from the disease [5,9,10,11,22]. Moreover, the implications of the effects of COVID-19 on the aerobic fitness of elite athletes upon return-to-play are still not well understood. Therefore, this systematic review and meta-analysis study aimed to evaluate the influence of COVID-19 on aerobic fitness in elite athletes after infection. The study was conducted with the following aims: (i) to describe the dynamics of $\dot{V}$O_2_max in post-COVID-19 athletes, depending on the symptoms of the disease; (ii) to assess athletic performance after 12 weeks of infection; and (iii) to discuss the pathways through which COVID-19 may influence $\dot{V}$O_2_max dynamics in these athletes.

## 2. Materials and Methods

### 2.1. Search Strategy

This systematic review and meta-analysis included prospective and retrospective observational studies published in the English language before 17 July 2023. The review was reported following Preferred Reporting Items for Systematic Reviews and Meta-analyses (PRISMA) reporting guidelines and was registered, respectively, on PROSPERO (CRD42023456799). Papers were screened from the PubMed database using the following keywords: (“COVID-19” OR “SARS-CoV-2”) AND (“maximal oxygen uptake” OR “$\dot{V}$O_2_max” OR “peak VO_2_”) AND (“athlete”).

### 2.2. Inclusion and Exclusion Criteria

The established inclusion criteria were the following: publications of reported case, cross-sectional, cohort, and case–control studies that performed CPET for measurement of $\dot{V}$O_2_max in post-COVID-19 elite athletes, regardless of sport modality, whether aerobic or anaerobic. An elite athlete was defined as an individual that had one or more of the following characteristics: high VO_2_ at the anaerobic threshold (AT) and $\dot{V}$O_2_max values (around 50 mL/kg/min for men and 40 mL/kg/min for women, depending on the sports category), running economy, anthropometric characteristics, participation in high-level competitions, and training characteristics (intensity, frequency, and duration) [24]. In addition, studies that evaluated athletes of any sport modality who had SARS-CoV-2 infection confirmed by polymerase chain reaction (PCR) or rapid antigen test and that could have asymptomatic, mild, or persistent symptoms (up to 12 weeks after infection) were also selected. Mild symptoms were defined as those who did not require hospitalization during the acute phase of the disease and without complications for more than 14 days, such as ageusia, anosmia, cough, diarrhea, fatigue, fever, headache, nasopharyngeal congestion, nausea, sore throat, and vomiting [25]. Papers involving intervention, lockdown, screening, face mask effect, or other contents unrelated to the infection and studies enrolling children or adolescent athletes were excluded from this review.

### 2.3. Assessment of Risk of Bias

The information of the included studies was extracted and independently analyzed by two reviewers, based on the Strengthening the Reporting of Observational Studies in Epidemiology (STROBE), for quality screening of cohort, case–control, and cross-sectional studies (combined) checklist [26], and the score above 50% was approved for this systematic review [14]. In addition, a comparison of the $\dot{V}$O_2_max means between treadmill or cycle ergometer methods for CPET was performed using Student’s *t*-test for independent samples to mitigate the risk of bias. Any disagreement found in the inclusion of studies and in the $\dot{V}$O_2_max mean comparison analysis was resolved by a third investigator.

### 2.4. Data Extraction and Analysis

After the selection of the suitable papers and the complete reading, the following information was synthesized: source, study type, study population (age and body mass index (BMI, Kg/m^2^) average, sex distribution, sport modality, and competitive level), number of post-COVID-19 and non-infected athletes, disease degree (asymptomatic, mild, or persistent symptoms), methods used for CPET (treadmill or cycle ergometer), the timing of evaluation since infection (days), $\dot{V}$O_2_max (mL/kg/min), and performed analytic comparisons in each study. These data were synthesized in an Excel spreadsheet by one reviewer and verified by two other independent reviewers.

The analyzed pooled athletes from the extracted studies were divided into two groups: post-COVID and without infection. Post-COVID-19 status was confirmed by PCR or rapid antigen test. Athletes who were uninfected and those who were infected but had information on $\dot{V}$O_2_max levels by CPET methods before infection were included in the infection-free group. The same athlete was included in both groups (post-COVID-19 and without infection) when the included studies compared his $\dot{V}$O_2_max level before and after the infection [5,8,9,10,11,16,22,25,27,28,29,30].

Additionally, the post-COVID-19 group was stratified according to the disease’s symptoms: asymptomatic, mild, or persistent symptoms. Persistent symptoms were defined as lasting more than 12 weeks after infection. Comparisons of means between the groups were performed using the independent Student’s *t*-test or one-way ANOVA test with a 95% confidence interval (95% CI). For three studies that only reported the median and interquartile range (IQR), the range between the median and upper and lower bounds was found to be homogenous (presumed normal distribution), so the median was appraised as the mean. This review categorized the $\dot{V}$O_2_max value (mL/kg/min) between females and males aged between 20 and 29 years, respectively, as very poor (≤30.9 and ≤36.7), poor (32.3–35.2 and 36.7–41.0), fair (36.0–38.5 and 41.7–44.6), good (39.5–42.4 and 45.4–48.4), and excellent (≥43.9 and ≥51.1), according to the Health-Related Physical Fitness Assessment Manual of the American College of Sports Medicine (ACSM) [31]. A linear regression was performed to assess the relationship between $\dot{V}$O_2_max values and BMI, and the moment of the evaluation since the infection among the subgroups. All the analyses were performed using IBM SPSS 20.0 Statistics for Windows (SPSS Inc., Chicago, IL, USA), and the level of statistical significance was defined as *p*-value < 0.05.

A mechanistic model of putative pathways underlying the influence of COVID-19 in $\dot{V}$O_2_max dynamics among elite athletes was proposed. The figure was produced with BioRender: Scientific Image and Illustration Software (https://biorender.com/, accessed on 18 October 2023).

## 3. Results

**Figure 1:**
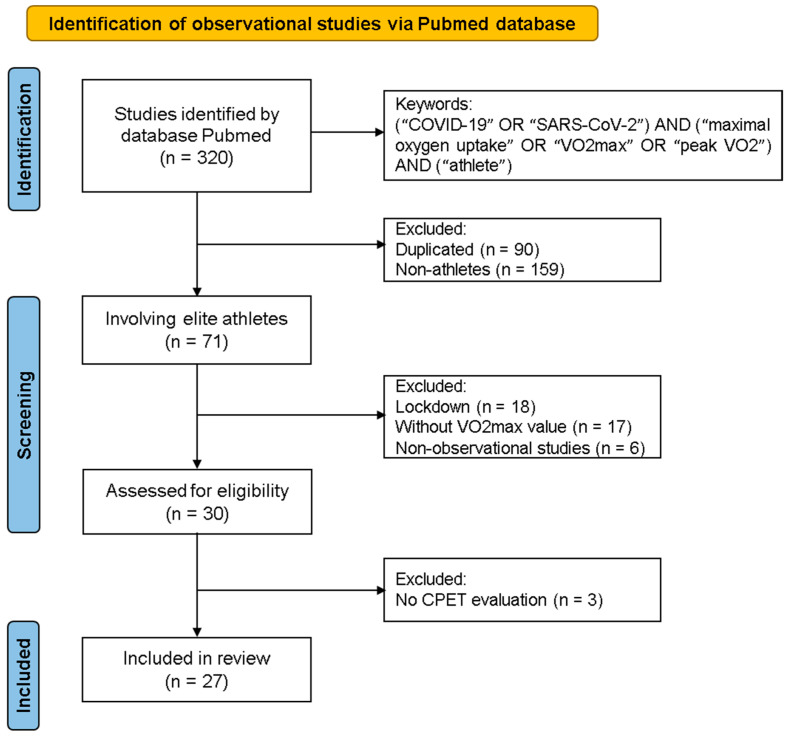
Flowchart of observational studies included in the systematic review and meta-analysis.

Of the 320 studies found in the PubMed database according to the proposed search strategy, 71 were performed with elite athletes. Among those, however, only 30 were assessed for full-text eligibility after reading the title and abstract. Finally, 27 studies were included in this systematic review, of which 21 were comparison studies and 6 were reported cases (Figure 1). Of note, three studies were conducted with the same population [25,29,30] so data were only extracted from one of them. Although all the studies were performed with elite athletes, eight articles also evaluated competitive non-elite athletes [6,7,25,27,29,30,32], and Wernhart et al. (2023) performed a comparison between elite athletes and recreational athletes [13].

Table 1 describes the baseline information (country, study design, number of athletes, and CPET evaluation) and the methodological quality of the included studies in this systematic review. A total of 2625 athletes (1464 post-COVID-19) of the included studies performed CPET assessment via either treadmill (*n* = 11; 40.7%) or cycle ergometer (*n* = 11; 40.7%) or both (*n* = 6; (22.2%). Although the case study of Daems et al. (2022) did not report on the CPET method used to assess $\dot{V}$O_2_max [21], it was in accordance with the established inclusion criteria. Importantly, there was no difference in the mean $\dot{V}$O_2_max depending on the method used for CPET (Student’s *t*-test, *p* = 0.33). All papers showed a percentage greater than 70% of study quality according to the STROBE checklist.

The average age of the pooled athletes was 25.7 ± 7.3 (18–56) years, with post-COVID-19 athletes being 5.6 ± 2.5 years older than those without infection (Student’s *t*-test, *p* = 0.03; 95% CI = 0.5–10.8). The evaluated athletes were of endurance, power, mixed, or skill sports modalities. The mean CPET evaluation timing since the infection was 75.7 ± 77.4 (2–270) days, while the VO_2_ at the AT was 33.8 ± 8.4 (22–49) mL/Kg/min. The demographic, anthropometric measures, sport modality, and oxygen consumption information of the pooled athletes are described in Table 2. In addition, the values of $\dot{V}$O_2_max and $\dot{V}$O_2_ at the AT between the groups with and without COVID-19 are described in Supplementary Table S1. A total of 21 selected studies were performed with symptomatic athletes, of which 12 presented mild [16,22,25,28,29,30,33,34,35,36,37,38] and the remaining 9 with persistent symptoms [8,9,10,11,12,13,16]. The studies found different persistent symptoms depending on the population studied. The most common were chest pain, cough, diminished capacity and exercise-induced shortness of breath, fatigue, myalgia, palpitations, peripheral skin symptoms, and tachycardia [6,7,8,9,11,13,16,27,39]. Three studies were only with asymptomatic [23,27,40], while three evaluated asymptomatic and athletes with persistent symptoms [12,32,39].

**Figure 2:**
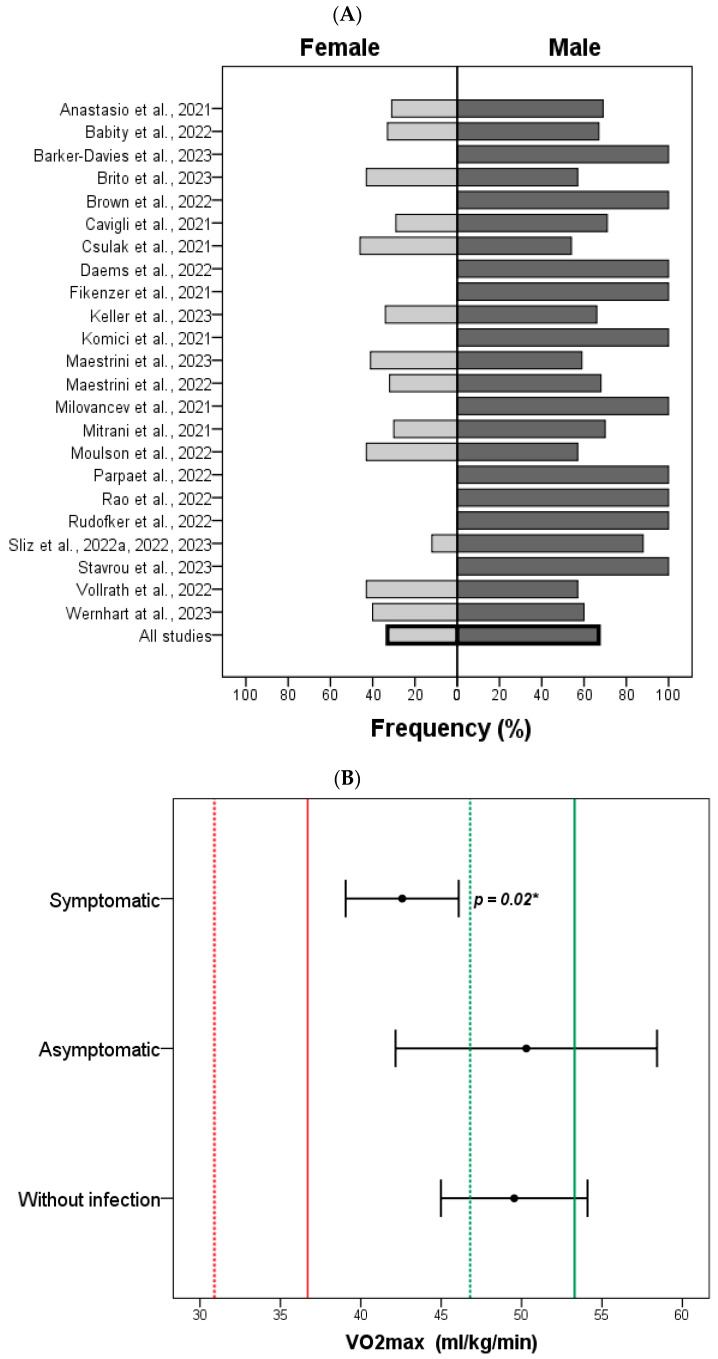
(**A**) Frequency of male and female athletes analyzed in each study. (**B**) Comparison of mean $\dot{V}$O_2_max among symptomatic and asymptomatic post-COVID-19 athletes and those without infection in the pooled analysis of the included studies. Most of the pooled athletes were males. The green line corresponds to an excellent level (female: 46.8 mL/kg/min and male: 53.3 mL/kg/min) and the red line to a very poor level (female: 36.7 mL/kg/min and male: 30.9 mL/kg/min) of aerobic fitness at aged 20–29 years, according to American College Sports Medicine (ACSM). * *p*-value was obtained by one-way ANOVA test.

Although some observational studies found no significant differences in the $\dot{V}$O_2_max levels between infected and uninfected athletes [5,6,7,28,37,38,39,40], the pooled analysis showed a lower $\dot{V}$O_2_max among post-COVID-19 athletes, mainly with persistent symptoms, when compared to those without infection (*p* = 0.03). Comparisons of the mean $\dot{V}$O_2_max among subgroups (infection-free, asymptomatic, mild, and persistent symptoms) are shown in Table 3. There was no significant difference observed when evaluating the recovery of $\dot{V}$O_2_max based on the timing of evaluation since infection (i.e., 0–30 days, 30–120 days, 120–240 days, >240 days (*p* = 0.31)). Of the 10 studies that performed analysis of $\dot{V}$O_2_max before and post-COVID, the mean value decreased by 6 mL/Kg/min post-infection. There was, however, no significant difference compared to CPET performed before infection. Figure 2 shows the comparison of subgroups according to fitness categories for $\dot{V}$O_2_max between females and males by age [31]. Athletes who had mild or persistent symptoms had a lower $\dot{V}$O_2_max than those without infection (Tukey test, *p* = 0.02). Nevertheless, according to the ACSM, the $\dot{V}$O_2_max value post-COVID-19 did not reach the very poor threshold (<15%), considering the age of 20 to 29 years for males (36.7 mL/Kg/min) and females (30.9 mL/Kg/min). It should be highlighted that approximately 70% of the sample studied in the observational studies included in this systematic review were males, except for the studies by Nedeljkovic et al. (2021) and Stojmenovic et al. (2023), who did not report the sex distribution of the evaluated athletes [8,23].

**Table 1:** Summary of observational studies that included cardiopulmonary exercise testing (CPET) post-COVID-19 in elite athletes.

Source	Origins	Study Design	Nº Infected/All Athletes	CPET Evaluation	Primary Analytic Comparisons	Quality Score (%) ^a^
Anastasio et al., 2021 [40]	Italy	Case–control study	13/26	Treadmill	Athletes recovered from mild–moderate COVID-19 were matched with elite cross-country skiers without SARS-CoV-2 infection	79.3
Babity et al., 2022 [27]	Hungary	Cross-sectional study	183/183	Treadmill	CPET comparisons were performed in 62 asymptomatic elite athletes between examination before and after the SARS-CoV-2 infection	82.8
Barker-Davies et al., 2023 [5]	United Kingdom	Case study	1/1	Cycle ergometer	CPET comparisons between 15 months prior and 5 months post-COVID-19 diagnosis	NA ^b^
Brito et al., 2023 [39]	Brazil	Cross-sectional study	46/46	Treadmill	CPET comparisons between symptomatic and asymptomatic after the SARS-CoV-2 infection in all study participants	82.8
Brown et al., 2022 [16]	USA	Case study	1/1	Cycle ergometer	CPET comparisons between 6 days before and 19 weeks after COVID-19 diagnosis	NA ^b^
Cavigli et al., 2021 [32]	Italy	Prospective study	90/90	Cycle ergometer	CPET comparison between asymptomatic vs. symptomatic individuals	79.3
Csulak et al., 2021 [28]	Hungary	Prospective study	14/46	Treadmill	CPET comparisons between infected and non-infected swimmers on pre- and post-COVID-19 pandemic	79.3
Daems et al., 2022 [21]	Netherlands	Case study	1/1	No information	Descriptive	NA ^b^
Fikenzer et al., 2021 [22]	Germany	Cross-sectional study	8/12	Cycle ergometer	CPET comparisons between the summer 2020 (t0) preparation phase and either after SARS-CoV-2 infection in winter 2020 or during routine control in winter 2020 (t1).	79.3
Keller et al., 2023 [33]	Germany	Cross-sectional study	157/1200	Treadmill or cycle ergometer	CPET comparisons of athletes with and without former COVID-19 infection	82.8
Komici et al., 2021 [6]	Italy	Case–control study	24/35	Treadmill	CPET comparisons of COVID-19 athletes and a group of competitive athletes following previous physical capacity evaluation after summer holidays and before starting training program tested negative for COVID-19	72.4
Maestrini et al., 2023 [34]	Italy	Cross-sectional study	219/219	Cycle ergometer	Descriptive	76.7
Maestrini et al., 2022 [35]	Italy	Cross-sectional study	47/47	Cycle ergometer	Descriptive	82.8
Milovancev et al., 2021 [36]	Serbia	Cross-sectional study	16/16	Treadmill	Descriptive	72.4
Mitrani et al., 2021 [37]	USA	Cross-sectional study	174/174	Treadmill	Descriptive	72.4
Moulson et al., 2022 [7]	USA	Prospective cohort study	21/63	Treadmill	Post-COVID athletes were compared with a matched reference group of healthy athletes without COVID-19 infection	83.9
Nedeljkovic et al., 2021 [8]	Serbia	Case study	1/1	Treadmill	CPET comparisons between 2-week and 3-month follow-up after infection with testing performed before infection	NA ^b^
Parpa et al., 2022 [9]	Cyprus	Cross-sectional study	21/21	Cycle ergometer	CPET comparisons between the same athletes before and after SARS-CoV-2 infection	72.4
Rao et al., 2022 [10]	USA	Case study	1/1	Cycle ergometer	CPET comparisons between before and after SARS-CoV-2 infection	NA ^b^
Rudofker et al., 2022 [11]	USA	Case study	1/1	Cycle ergometer	Descriptive	NA ^b^
Śliż et al., 2022 [25]; Śliż et al., 2022 [29]; Śliż et al., 2023 [30]	Poland	Cross-sectional study	49/49	Treadmill or cycle ergometer	CPET comparisons between before and after SARS-CoV-2 infection	82.8
Stavrou et al., 2023 [38]	Greece	Cross-sectional study	20/40	Treadmill	CPET comparisons between previously infected with SARS-CoV-2 versus non-infected athletes	79.3
Stojmenovic et al., 2023 [23]	Serbia	Prospective study	220/220	Treadmill	CPET comparisons among the three strains of the SARS-CoV-2 (Wuhan, Delta, or Omicron)	75.9
Vollrath et al., 2022 [12]	Germany	Prospective study	60/60	Cycle ergometer	CPET comparisons between t0 (4.1 ± 3.8 months after infection) and t1 three months later (3.3 ± 0.5 months)	79.3
Wernhart et al., 2023 [13]	Germany	Cross-sectional study	83/83	Cycle ergometer	CPET comparisons between elite and recreational athletes who reported persistent symptoms of COVID-19 infection	82.8

^a^ The score was based on the STROBE checklist of cohort, case–control, and cross-sectional studies (combined). ^b^ Reported case studies that do not have STROBE assessment; however, they were included in this systematic review because they were considered relevant. NA, not applicable.

**Table 2:** Sample characteristics and oxygen consumption post-COVID-19 observed in each study.

Source	Age (Years) ^a^	Male Sex N (%)	BMI (Kg/m^2^) ^a^	Sport Modality	Timing of Evaluation Since Infection (Days) ^a,b^	$\dot{V}$O_2_max Post-Infection (mL/kg/min) ^a^	VO_2_ at AT Post-Infection (mL/kg/min) ^a,d^
Anastasio et al., 2021 [40]	Cases: 21 ± 5 Controls: 20 ± 4	Cases: 10 (77) Controls: 8 (62)	Cases: 22 ± 2 Controls: 21 ± 1	Cross-country skiing	28–42	56.9 (48.5–64.3)	48.8 (43.5–56.0)
Babity et al., 2022 [27] ^c^	20 (17–24)	122 (74)	No information	Basketball; ice hockey; water polo; wrestling; swimming; running; football; handball; others	94 (67–130)	50.9 ± 6.0	44.2 ± 5.0
Barker-Davies et al., 2023 [5]	30	1 (100)	No information	Distance running	35	59	27.0
Brito et al., 2023 [39]	30 ± 9	26 (57)	26 ± 5	Soccer; CrossFit; rugby; athletics; para athletic; combat sports; swimming; volleyball; rowing; others	14–252	41.1 ± 8.7	23.5 ± 6.2
Brown et al., 2022 [16]	23	1 (100)	No information	No information	133	38.8	No information
Cavigli et al., 2021 [32]	24 ± 10	64 (71)	23 ± 3	Endurance; mixed; power; skill	No information	39.0 ± 6.6	No information
Csulak et al., 2021 [28]	Cases: 23 ± 4 Non-infected: 24 ± 4	Cases: 7 (50) Non-infected: 18 (56)	No information	Swimming	10–14	Female: 52.9 ± 4.1 Male: 56.5 ± 4.9	No information
Daems et al., 2022 [21]	21	1 (100)	No information	Soccer	270	45	No information
Fikenzer et al., 2021 [22]	Cases: 27 ± 4 Non-infected: 22 ± 3	12 (100)	Cases: ~27 (97 kg; 191 cm) Non-infected: ~26 (96 kg; 194 cm)	Handball	20	Cases: ~39.2 (3790 ± 513 mL/min; 96.7 ± 5.4 kg)	No information
Keller et al., 2023 [33]	Cases: 23 ± 7Non-infected: 22 ± 12	Cases: 122 (78) Controls: 667 (64)	Cases: 24 (22–26) Non-infected: 22 (20–24)	Several	No information	43.4 (38.3–48.0)	NA
Komici et al., 2021 [6]	Cases: 24 (20–26) Non-infected: 21 (10–24)	35 (100)	Cases: 23 (22–24) Non-infected: 23 (22–26)	Soccer	10–30	50.1 (47.7–51.6)	No information
Maestrini et al., 2023 [34]	23 (19–27)	129 (59)	23 (22–25)	Endurance; mixed power; skill	10 (6–17)	39 ± 8	No information
Maestrini et al., 2022 [35]	26 ± 4	32 (68)	24 ± 3	Endurance; power; mixed	9 (6–13)	42 ± 6	22 ± 4
Milovancev et al., 2021 [36]	24 ± 5	16 (100)	24 ± 2	Volleyball	20 ± 5	44.1 ± 3.4	40.8 ± 3.9
Mitrani et al., 2021 [37]	21 (19–22)	122 (70)	No information	Baseball; football; basketball; volleyball; soccer; swimming; others	19 (16–25)	37.7 ± 8.0	No information
Moulson et al., 2022 [7]	Cases: 22 ± 4 Non-infected: 22 ± 4	Cases: 12 (57) Non-infected: 24 (51)	Cases: 23 ± 3 Non-infected: 24 ± 3	Endurance; team sport; mixed	90 ± 60	44.6 ± 9.1	35.7 ± 11.3
Nedeljkovic et al., 2021 [8]	32	No information	No information	No information	15	32.2	23.1
Parpa et al., 2022 [9]	24 ± 6	21 (100)	~23 (74 ± 5 kg; 178 ± 5 cm)	Soccer	60	54.3 ± 5.2	36.9 ± 5.2
Rao et al., 2022 [10]	18	1 (100)	No information	Rowing	No information	28	No information
Rudofker et al., 2022 [11]	56	1 (100)	No information	No information	270	33.1	No information
Śliż et al., 2022 [25]; Śliż et al., 2022 [29]; Śliż et al., 2023 [30]	40 ± 8	43 (88)	24 ± 3	Endurance	155 ± 82	45 ± 7	32 ± 6
Stavrou et al., 2023 [38]	Cases: 25 ± 4 Non-infected: 25 ± 4	Cases: 20 (100) Non-infected: 20 (100)	Cases: 23 ± 2 Non-infected: 24 ± 1	Soccer	2	55.7 ± 4.4	No information
Stojmenovic et al., 2023 [23]	Soccer: 23 ± 5 Basketball: 25 ± 5	No information	No information	Soccer; basketball	22	Wuhan: 47.6 ± 5.1 Delta: 47.9 ± 4.6 Omicron: 50.6 ± 4.1	Wuhan: 26.2 ± 4.7 Delta: 24.9 ± 3.7 Omicron: 30.4 ± 4.4
Vollrath et al., 2022 [12]	35 ± 12	34 (57)	24 ± 4	Several	120 ± 90	t0: Symptoms free: 44.7 ± 7.7 Persistent symptoms: 33.7±9.9	No information
Wernhart et al., 2023 [13]	Elite: 22 ± 4 Recreational: 35 ± 13	Elite: 29 (67) Recreational: 21 (53)	Elite: 24 ± 2 Recreational: 24 ± 4	Football; handball; endurance; badminton; swimming	180	Elite: 44.8 ± 6.8	No information

Mixed corresponds to the sports whose participants are not of a single sex. Several correspond to different sports. ^a^ The values were presented as absolute value, mean ± standard deviation, median (IQR), or range. ^b^ One week was considered with 7 days, while one month with 30 days. ^c^ Information obtained from 165 asymptomatic athletes. ^d^ VO_2_ at AT post-infection = oxygen uptake at the AT after the COVID-19 infection. AT, anaerobic threshold; BMI, body mass index; NA, not applicable; $\dot{V}$O_2_max, maximal oxygen uptake.

**Table 3:** Comparison of the mean $\dot{V}$O_2_max between post-COVID-19 and infection-free, as well as asymptomatic and symptomatic athletes of all included studies.

Comparisons	Mean ± SD	*p*-Value ^a^	Post Hoc Test (Tukey HRD)	Mean Difference (95% CI)
Without infection	49.54 ± 8.56	0.03	---	1 ^b^
Post-infection	43.90 ± 7.92			5.64 (0.52–10.76)
Without infection	49.54 ± 8.56	0.06	1 ^b^	1 ^b^
Asymptomatic	50.29 ± 5.11		0.99	−0.75 (−13.01–11.51)
Mild symptoms	43.70 ± 6.11		0.27	5.84 (−2.74–14.44)
Persistent symptoms	41.55 ± 9.79		0.07	8.00 (−0.38–16.37)
Without infection	49.54 ± 8.56	0.05	1 ^b^	1 ^b^
Asymptomatic or Mild symptoms	45.45 ± 6.43		0.36	4.09 (−3.14–11.32)
Persistent symptoms	41.55 ± 9.79		0.04	7.99 (0.31–15.68)
Without infection	49.54 ± 8.56	0.02	1 ^b^	1 ^b^
Asymptomatic	50.29 ± 5.11		0.98	0.75 (−11.79–10.29)
Symptomatic	42.57 ± 8.13		0.03	6.97 (0.54–13.39)
Asymptomatic	50.29 ± 5.11	0.08	----	1 ^b^
Symptomatic	42.57 ± 8.13			7.71 (−1.02–16.45)
Asymptomatic	50.29 ± 5.11	0.18	1 ^b^	1 ^b^
Mild symptoms	43.70 ± 6.11		0.34	6.60 (−4.96–18.15)
Persistent symptoms	41.55 ± 9.79		0.16	8.75 (−2.68–20.17)

^a^ *p*-value was obtained by Student’s *t*-test or one-way ANOVA. ^b^ Reference value. CI, confidence interval; SD, standard deviation.

**Figure 3:**
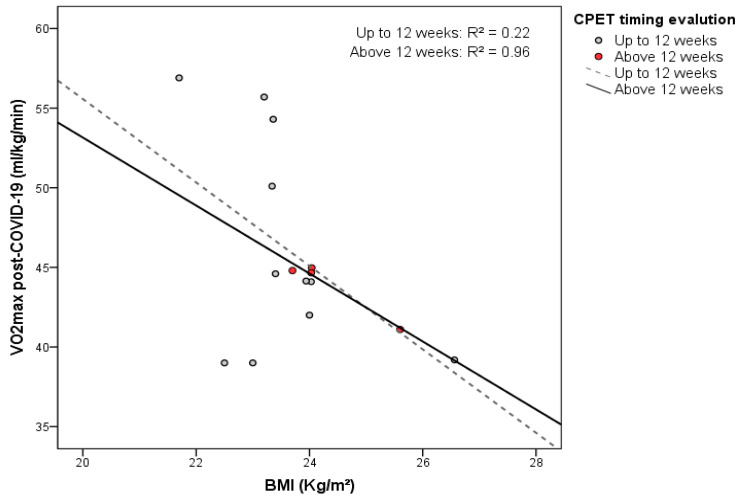
Relation between $\dot{V}$O_2_max post-COVID-19 and BMI according to CPET evaluation timing before and after 12 weeks. Athletes with CPET evaluation above 12 weeks showed a significant negative correlation between the variables (*p* = 0.01). *p*-value was obtained by the one-way ANOVA test.

There was no significant difference in BMI between the post-COVID-19 and infection-free athletes (23.8 ± 1.1 Kg/m^2^ versus 23.4 ± 1.4 Kg/m^2^, respectively). However, the $\dot{V}$O_2_max and BMI were inversely correlated. For each increase of 1 Kg/m^2^ in BMI, the mean $\dot{V}$O_2_max significantly decreased by 2.9 mL/Kg/min in the post-infected subgroup (one-way ANOVA test, *p* = 0.04), while there was no difference in athletes without infection. Moreover, residual analysis of the linear regression model showed normal distribution and homoscedasticity between $\dot{V}$O_2_max post-COVID-19 of the athletes who performed CPET above 12 weeks and independent variables (age and BMI). Thus, BMI contributed five times more than age to lower $\dot{V}$O_2_max (β: −1.0; 95% CI: −2.7–−1.6 versus β: 0.2; 95% CI: 0.1–1.1, respectively) in athletes who performed CPET after 12 weeks post-COVID-19. In contrast, there was no correlation among those who rated the 12-week mark (Figure 3).

**Figure 4:**
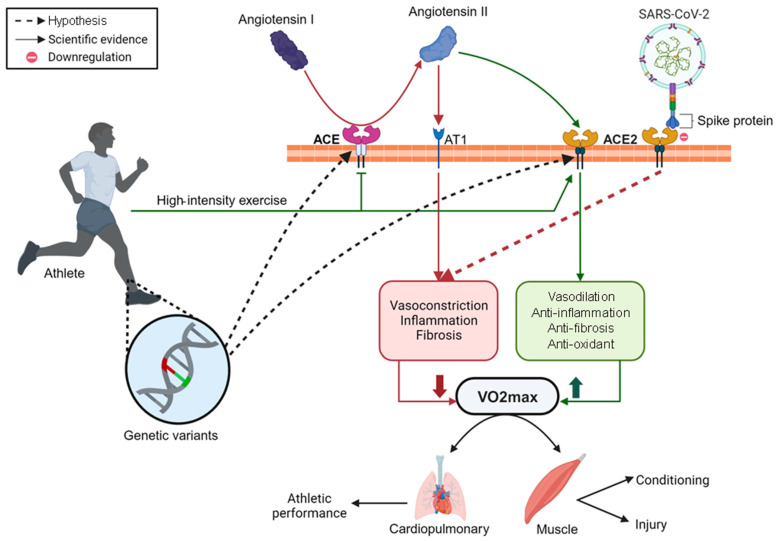
Hypothesized mechanism of vasoconstriction/vasodilation imbalance in $\dot{V}$O_2_max dynamics and performance of elite athletes post-COVID-19. The green line corresponds to the pathway that increases $\dot{V}$O_2_max, while the red line is the one that decreases $\dot{V}$O_2_max.

Based on these findings and the biological importance of the effect of $\dot{V}$O_2_max dynamics has in elite athletes, we hypothesized a possible relationship between the COVID-19 disease and athlete performance (Figure 4).

## 4. Discussion

In this systematic review and meta-analysis study, a total of 27 observational studies performed CPET on 1464 post-COVID-19 athletes, including approximately 570 with mild symptoms and 400 with persistent symptoms up to 12 weeks after infection. Vollrath et al. (2022) observed the presence of persistent symptoms in 73.3% of the 60 athletes evaluated four months after infection, and in 62% at three months later [12]. In addition, a 21-year-old male football player, with mild symptoms during infection, began experiencing palpitations one day after returning to training and persisted for one month. Following cardiac magnetic resonance imaging, he was diagnosed with focal COVID-19 myocarditis [21].

Physical fitness reduces the severity of upper respiratory tract infections [41]. However, SARS-CoV-2 infection impairs lymphopoiesis [42], which may contribute to a more severe disease course in high-performance athletes. A cross-sectional study conducted prior to vaccination found that 26% of athletes who did not undergo diagnostic testing for COVID-19 reported at least three mild symptoms of the illness during the lockdown, while only 11% of those infected were asymptomatic and 1.5% were hospitalized for illness [43], making the return to practice after infection a concern. A challenge for sports medicine is to differentiate between cardiopulmonary disease and common flu-like symptoms after vigorous exercise in elite athletes [5,44]. In endurance athletes, the immune system can be suppressed within 12 h of high-intensity exercise or for 2 weeks after a marathon race [45]. In addition, exercise alters the number of circulating lymphocytes and the release of cortisol, which affects the expression and function of anti-inflammatory cytokines [46]. Myocarditis is one of the main complications of viral syndromes, and COVID-19 may increase the incidence of this cardiac injury [17,47]. COVID-19 causes a more severe and prolonged reduction in $\dot{V}$O_2_max, particularly when persistent symptoms are present. Mitrani and colleagues (2022) observed an incidence of post-COVID-19 myocardial involvement of 2.9%, detected around 18.5 days after a positive COVID-19 PCR diagnosis among highly trained athletes, even with normal $\dot{V}$O_2_max [37].

A study by Hull et al. (2022) found that athletes with COVID-19 experienced a median symptom duration of 10 days and training loss ranging from 12 to 30 days, significantly exceeding the 6 (0–7) days of training loss associated with non-COVID-19 respiratory illnesses [48]. Exercise intolerance has been frequently reported as a post-COVID sequelae, especially in individuals hospitalized during the acute infection period [49]. Although the frequency of hospitalization for COVID-19 in athletes is less than 2% [50], our analysis of the pooled studies showed lower cardiorespiratory fitness in post-COVID-19 athletes compared to infection-free athletes, and those with persistent symptoms exhibited lower $\dot{V}$O_2_max levels. This suggests that even elite athletes, who typically have a higher aerobic capacity compared to sedentary individuals, may experience significant respiratory and muscular strain during exercise [38], leading to performance limitations. Studies have demonstrated that SARS-CoV-2 infection reduced the $\dot{V}$O_2_max levels and anaerobic threshold, resulting in increased respiratory work during exercise [38,40,49]. An elite long-distance runner with a reduction in anaerobic transition showed a 27 W decrease in workload and a 13% decrease in oxygen uptake, representing a significant loss in aerobic capacity [5]. In addition, a meta-analysis conducted by Durstenfeld and colleagues (2022) found a difference of approximately 5 mL/kg/min less in adult athletes and non-athletes with persistent symptoms compared to recovered individuals. However, 53% were hospitalized patients with an average age of 50 years [51]. Existing evidence indicates that $\dot{V}$O_2_max levels oscillate according to age, increasing up to the 40–49 age group and decreasing in older individual non-athletes (>50 years) [52]. Although elite athletes are younger, starting their sporting career at around 10 years old and retiring at 30 [53], athletes under 27 years old were associated with a three-fold higher risk of infection [43].

High-intensity training can improve $\dot{V}$O_2_max by up to 15% in elite athletes [54]. Even with different training protocols (e.g., high-intensity interval training, functional training) and differences in the dynamics of sport modalities (e.g., endurance, power, skill), cardiorespiratory fitness is significantly higher in elite athletes compared with non-elite athletes [55] and non-athletes [56]. Wernhart and colleagues (2023) analyzed the cardiopulmonary profile of elite and recreational athletes with post-exercise malaise after COVID-19 infection. They found that the recreational athletes had a $\dot{V}$O_2_max level of 12 mL/kg/min lower compared to their counterparts. In addition, 70% of recreational athletes had an inadequate cardiopulmonary response, compared to 40% of elite athletes [13]. In this systematic review, most of the analyzed pooled athletes were males aged 20–29 years old, and the average $\dot{V}$O_2_max in those with persistent symptoms was around 42 mL/kg/min, not reaching the poor grade (<35% aerobic fitness), according to ACSM for this category [31]. This may explain why elite athletes had fewer hospitalizations and required mechanical ventilation.

Well-known risk factors for infection and disease severity included age, male sex, obesity, and comorbidities [43,57]. The virus enters host cells by binding its spike protein (S) with the angiotensin-converting enzyme 2 (ACE2), one of the main enzymes of the renin–angiotensin system (RAS). This receptor is expressed in the cell membrane of the oral mucosa, respiratory tract, heart cells, adipocytes, and muscle [58]. Functionally, ACE2 is responsible for the hydrolysis of angiotensin II into angiotensin 1–7, which increases vasodilation, and produces anti-inflammatory, antioxidant, and anti-fibrosis effects [59]. The viral protein spike downregulates ACE2, causing vasoconstriction, increasing the inflammatory response [60], and decreasing the $\dot{V}$O_2_max value in athletes [5,9,10,11,16,22]. The expression of the ACE2 gene is reported to be positively correlated with increased BMI in COVID-19 subjects. Specifically, ACE2 overexpression is observed in individuals with a BMI of more than 30 kg/m^2^ [61]. Our pooled analysis of the athletes revealed a decrease of almost 3 mL/Kg/min in $\dot{V}$O_2_max levels after the acute course of COVID-19 and of 1 mL/kg/min 12 weeks later according to the increase in the BMI. The athlete’s fast recovery after COVID-19 can be explained by the rehabilitation medicine protocol during the return to training, which can reestablish $\dot{V}$O_2_max as before the infection [5,11,21,27]. However, a higher ACE2 expression might contribute to lower $\dot{V}$O_2_max levels in athletes with persistent symptoms.

Based on the findings, we propose a hypothesis of how COVID-19 disease may influence the vasoconstriction/vasodilation imbalance and affect $\dot{V}$O_2_max dynamics in elite athletes (Figure 4). Briefly, high-intensity exercise interacts with RAS, inducing a downregulation of the angiotensin-converting enzyme (ACE) and upregulation of ACE2, which causes vasodilation [58,60], and improves the $\dot{V}$O_2_max [60]. Recent studies have shown that genetic variants can influence $\dot{V}$O_2_max dynamics and aerobics [20,60]. Thus, single-nucleotide polymorphisms (SNPs) in combination with environmental factors (e.g., exercise capacity and exercise intensity) may modulate the $\dot{V}$O_2_max phenotype and contribute to oxygen delivery [62]. The genetic influence on $\dot{V}$O_2_max is still not fully understood; however, the *ACE* gene has been associated with higher $\dot{V}$O_2_max levels and better endurance performance in well-trained athletes [63]. Furthermore, SNPs on *ACE* and *ACE2* genes were associated with musculoskeletal injuries in athletes [64], and recently with infection and the severity of COVID-19 in the general population [65]. For example, genetic variants in specific targets, such as ACE and ACE2 SNPs, may impair the performance and muscular endurance of athletes and affect their return-to-play performance after COVID-19, which may also negatively affect their mental health. The absence of training during the COVID-19 pandemic has already been associated with the risk (~three-fold) of depression in athletes [66], demonstrating that time away from games and championships can affect their quality of life and well-being during their athletic careers.

High levels of cardiorespiratory and muscular fitness are critical for athletes as they enhance athletic performance and reduce the risk of muscular injury. In high-performance sports, even small differences in $\dot{V}$O_2_max can have a significant impact on fitness outcomes [48]. Chen et al. (20-22) demonstrated that a reduction of 2.5 mL/kg/min in $\dot{V}$O_2_max during two weeks of detraining resulted in decreased muscle strength and induced hemodynamic and muscular adaptations [67]. A reduction of 6–8 mL/kg/min in $\dot{V}$O_2_max represents a significant decrease in aerobic capacity, with potential implications for endurance, recovery, and overall athletic performance. Although data in the literature indicate a satisfactory return of $\dot{V}$O_2_max three months after infection in competitive athletes, especially in mixed teams and asymptomatic individuals [27], returning to training with exhaustive and excessive exercise after SARS-CoV-2 infection can compromise cardiorespiratory function [68,69]. This results in a dysregulated systemic inflammatory response and a significant reduction in immune system function [70]. As elite athletes often feel pressure to return to training and competition as quickly as possible, the resumption of intense exercise may promote the development of persistent COVID-19 symptoms, even in those who were asymptomatic or experienced mild disease symptoms [68].

The data highlight concerns about the risk of long-term silent symptoms, even in athletes who experienced mild to moderate symptoms during COVID-19. Therefore, we recommend that the multidisciplinary sports health care team and coaches implement individualized return-to-training protocols, including a gradual progression in the frequency and intensity of activities, while avoiding high-intensity exercise initially. It is essential to monitor cardiorespiratory testing, such as CPET, to track aerobic conditioning throughout this process, allowing athletes to safely increase the training intensity without causing sequelae that could jeopardize their professional future [68].

Despite the promising findings, this study had some limitations that need to be addressed. Due to the inclusion of case studies and some analytical studies that presented the median rather than mean $\dot{V}$O_2_max value, it is unclear whether the significance found in the pooled analysis is representative of the athlete’s population. The absence of data on age, sex, and BMI in some of the included studies could represent possible selection bias and may have contributed to the exaggeration of the effect estimated in the linear regression analysis. A few athletes who were more severely affected by the infection may have had a longer recovery period without physical activity, resulting in lower VO_2_max values. Because some of the studies in this systematic review evaluated athletes from mixed modalities (aerobic and anaerobic), it was not possible to perform a stratified analysis, as this could introduce systematic error into our interpretation. In addition, because we are using aggregated data, stratification could potentially underestimate statistical power, thereby compromising the quality of the study. Furthermore, various studies were performed with elite and non-elite athletes [6,7,11,13,25,27,29,30,32], who most likely have different training protocols and outcomes during physical activity. However, a strength of this review is the relevant quality of the included observational studies, which achieved more than 70% of the STROBE checklist score. In addition, the pooled data from the included studies enable the analysis of a total of 2625 athletes who underwent CPET evaluation. Clinical variability in the persistent symptoms and the method used in CPET could have influenced the $\dot{V}$O_2_max levels and caused information bias in the analyzed data [50]. However, there was no difference between the treadmill and cycle ergometer methods in the studies included in this systematic review and meta-analysis.

## 5. Conclusions

This systematic review and meta-analysis found evidence that aerobic fitness is diminished after COVID-19. Although observational studies have reported that athletes recover their $\dot{V}$O_2_max quickly after infection, those who underwent CPET at more than 12 weeks showed reduced exercise capacity, especially those with a higher BMI. Thus, this pooled analysis supports the implementation of a management guide for elite and non-elite athletes post-COVID-19, whether asymptomatic or symptomatic, to promote health and well-being, and avoid the sequelae of exercise intolerance and sports injuries upon return to training and competitions.

## Data Availability

Not applicable.

## Supplementary Materials

The following supporting information can be downloaded at: https://www.mdpi.com/article/10.3390/sports13020040/s1, Supplementary Table S1: $\dot{V}$O_2_max values with or without COVID-19 for the comparison groups [5,6,7,8,9,10,11,12,13,16,22,27,28,30,32,33,38,39,40].

## Abbreviations

The following abbreviations are used in this manuscript:

ACE	Angiotensin-converting enzyme
ACE2	Angiotensin-converting enzyme 2
ACSM	American College of Sports Medicine
AT	Anaerobic threshold
ATP	Adenosine triphosphate
BMI	Body mass index
CI	Confidence interval
CPET	Cardiopulmonary exercise test
IQR	Interquartile range
PCR	Polymerase chain reaction
PRISMA	Preferred Reporting Items for Systematic Reviews and Meta-analysis
RAS	Renin–angiotensin system
S	Spike protein
SD	Standard deviation
SNP	Single-nucleotide polymorphism
STROBE	Strengthening the Reporting of Observational Studies in Epidemiology
$\dot{V}$O_2_max	Maximal oxygen uptake
