# Supplementary material for: A Systematic Review and Meta-Analysis on Aerobic Fitness Dynamics in Post-COVID-19 Athletes: Implications in the Return-to-Play Performance
Source: Sports (Basel). 2025 Feb 5;13(2):40. doi: 10.3390/sports13020040 (PMC11860767; doi:10.3390/sports13020040)
Supplement: Supplementary file 1 [file sports-13-00040-s001.zip › sports-3408346-supplementary.pdf]

Supplementary Table S1. V̇O2max and V̇O2 at AT values with or without COVID-19 for the comparison groups

Source	Comparisons	Follow-up between CPET evaluation (weeks)	V̇O2max max after infection (ml/kg/min)	V̇O2max max reference group (ml/kg/min)	V̇O2 at AT after infection (ml/kg/min)	V̇O2 at AT reference group (ml/kg/min)
Anastasio et al., 2021 [33]	COVID-infected vs non-infected athletes	NA	56.9	60	48.8	49
Babity et al., 2022 [27]	Before and after the infection	9	52.2	49.9	44.2	41.8
Barker-Davies et al., 2023 [5]	Before and after the infection	5	59	61	27	35
Brito et al., 2023 [34]	Symptomatic vs asymptomtic	NA	40.6	41.9	23.5	24.2
Brown et al., 2022 [16]	Before and after the infection	5	38.8	40.48	No information	No information
Cavigli et al., 2021 [32]	Symptomatic vs asymptomtic	NA	38.1	41.9	No information	No information
Csulak et al., 2021 [28]	Before and after the infection	24	54.7	54.1	No information	No information
Fikenzer et al., 2021 [22]	Before and after the infection	6	39.19	42.21	No information	No information
Fikenzer et al., 2021 [22]	COVID-infected vs non-infected athletes	6	39.19	40.74	No information	No information
Keller et al., 2023 [35]	COVID-infected vs non-infected athletes	NA	43.4	45.3	No information	No information
Komici et al., 2021 [6]	COVID-infected vs non-infected athletes	NA	50.1	49	No information	No information
Moulson et al., 2022 [7]	COVID-infected vs non-infected athletes	5	44.6	46.4	35.7	36

Nedeljkovic et al., 2021 [8]	Before and after the infection	3	32.2	31.4	23.1	22
Parpa et al., 2022 [9]	Before and after the infection	4	54.3	57.35	36.89	37.12
Rao et al., 2022 [10]	Before and after the infection	NA	28	49.1	No information	No information
Rudofker et al., 2022 [11]	Before and after the infection	NA	33.1	62.5	No information	No information
Śliż et al., 2022 [25]; Śliż et al., 2022 [29]; Śliż et al., 2023 [30]	Before and after the infection	50	44.97	47.81	32.35	34.96
Stavrou et al., 2023 [40]	COVID-infected vs non-infected athletes	NA	55.7	55.4	No information	No information
Vollrath et al., 2022 [12]	Follow-up SF-SF (t0-t1)	3	44.66	43.82	No information	No information
Vollrath et al., 2022 [12]	Follow-up SF-SF (t0-t1)	3	44.66	43.82	No information	No information
Vollrath et al., 2022 [12]	Follow-up PS-PS (t0-t1)	3	33.66	33.84	No information	No information
Vollrath et al., 2022 [12]	Follow-up PS-PS (t0-t1)	3	33.66	33.84	No information	No information
Wernhart et al., 2023 [13]	Elite vs non-elite - Persistent symptoms	NA	44.88	32.7	No information	No information

AT is Anaerobic threshold; CPET: Cardiopulmonary exercise testing; NA is Not applicable; VO_2 is maximal oxygen uptake.
